# Irreversible functional network deformation induced by preprocessing scheme order in multivariate EEG recordings

**DOI:** 10.3389/fninf.2026.1907920

**Published:** 2026-09-28

**Authors:** J. Daniel Arzate-Mena, Paola V. Olguín-Rodríguez, Maria Corsi-Cabrera, Erik A. López-Alvarado, Markus F. Müller

**Affiliations:** 1 Centro de Investigación en Ciencias, Universidad Autónoma del Estado de Morelos (UAEM), Cuernavaca, Morelos, Mexico; 2 Centro de Ciencias de la Complejidad (C3), Universidad Nacional Autónoma de México, Mexico City, Mexico; 3 Instituto de Neurobiología, Universidad Nacional Autónoma de México, Querétaro, Mexico; 4 Facultad de Medicina, Universidad Nacional Autónoma de México, Mexico City, Mexico; 5 Centro Internacional de Ciencias A. C., Cuernavaca, Morelos, Mexico

**Keywords:** EEG, EEG pre-processing, EEG reference, functional connectivity, multivariate signal analysis, spectral slope, spurious correlations

## Abstract

**Introduction:**

The analysis of Electroencephalographic recordings is non-trivial, the order in which filtering and re-referencing are applied during multivariate signal preprocessing has a critical and underappreciated consequence for functional network estimation.

**Methods:**

Using EEG sleep recordings from healthy subjects, synthetic data from controlled numerical models (Nf-Tori and IAAFT surrogates), and surrogate-based statistical testing, we provide a quantitative comparison of two preprocessing schemes that differ solely in the order of filtering and re-referencing (filtering followed by re-referencing—FR, and re-referencing followed by filtering—RF).

**Results:**

We show that the RF scheme induces spurious cross-correlations that increase systematically with the frequency band and are strongly modulated by the power spectral exponent β of the signal. Critically, for signals with steep power spectra (β ≥2), a regime that characterizes slow-wave sleep, epileptic post-ictal periods, anesthesia, neonatal, and patients in vegetative states, the RF scheme produces artificial correlations that are irreversible. They cannot be eliminated *post-hoc* even when surrogate-corrected cross-correlations (SAC-matrix) are applied. The mechanism underlying this effect is due to the high-amplitude slow-frequency components that dominate the median reference signal over extended time intervals, embedding redundant information across all channels. In contrast, the FR scheme introduces only small, systematic negative shifts in cross-correlations that are not statistically significant and can be effectively corrected using surrogate-based methods.

**Conclusions:**

We therefore recommend the FR scheme combined with surrogate-corrected cross-correlations for all applications involving signals with β ≥2. Although this finding is demonstrated in EEG, it applies to any multivariate recording where spatial re-referencing and band-pass filtering are applied sequentially.

## 1 Introduction

One of the most common techniques for acquiring empirical data from brain dynamics is electroencephalography (EEG). It is non-invasive, available in portable versions, and offers a high temporal resolution, which is advantageous for recording fast mental processes. However, measurement of brain dynamics bears notable difficulties, not only because of the non-stationary character, the extremely high dimension of the system, or the contamination with noise and muscle artifacts, but also because EEG recordings require the determination of a reference point, given that one is not able to measure the electrical potential itself but solely potential differences.

The problem of selecting a suitable reference scheme naturally depends on the characteristics on which to focus and has been extensively discussed in the literature (see e.g., Delorme, 2023; Valdes-Sosa et al., 2020). The extraction of univariate features, such as the power spectrum, or the estimation of bivariate measures, like cross-correlation or phase synchronization, may require different choices to minimize distortions caused by the reference scheme (Delorme, 2023; Hagemann et al., 2001; Rios et al., 2019; Rummel et al., 2007; Tian and Yao, 2013). Furthermore, filtering empirical recordings in narrow frequency bands may cause numerical problems, as shown in Rios-Herrera et al. (2016).

In the present contribution, we discuss the case where it is aimed to construct the functional network of brain activity, which is in a certain sense a kind of data pre-processing because in a further step, one usually extracts some topological properties of this network. Various techniques are used, such as graph theory (Barabási and Albert, 1999; Bullmor and Sporns, 2009; Watts and Strogatz, 1998), random matrix theory (Baier et al., 2007), variants of principal component analysis (Leonardi et al., 2012) and methods based on the theory of dynamic systems (Apablaza-Yevenes et al., 2024; Arzate-Mena et al., 2022; Müller et al., 2014; Olguín-Rodríguez et al., 2019). It is therefore of major importance to avoid significant distortions through the pre-processing scheme, such as the combination of selecting the most favorable EEG reference and signal filtering.

It seems that for each of the problems separately optimal solutions have been proposed and as could be expected, it appears that the answer depends on the specific application.

The authors of Rios et al. (2019) showed by using a variety of numerical models that allow controlling for a large number of different genuine cross-correlation patterns that the median reference causes less distortions in comparison to other popular reference schemes. This was recently confirmed in Marín–García et al. (2024) where it was demonstrated quantitatively that the functional network obtained using the median reference in EEG closely resembles that obtained in magnetoencephalography (MEG), which is reference-free. Thus, in the present work, we will concentrate on this reference scheme.

On the other hand, filtering empirical recordings in the classical EEG bands (or filtering into even narrower frequency bands, Barry et al., 2003; Doesburga et al., 2009) can also cause serious problems like the induction of spurious cross-correlation (Rios et al., 2019). Since for the median reference one of the active electrodes serves as a reference, it influences the entire grid at this instant of time. Although it seems that the functional network is less affected by that (Marín–García et al., 2024; Rios et al., 2019) this might have consequences for the power spectra of each recording, which in turn may contaminate the cross-correlation pattern by so called “random correlations” (Laloux et al., 1999; Plerou et al., 1999, 2002; Müller et al., 2005, 2006).

The present work aims to study the possible influence of the combination of re-referencing and filtering. Here we investigate the influence of two different pre-processing schemes on the topological properties of the functional network generated by linear cross-correlations. To this end, we analyze artificial data with well-defined uni- and bi-variate properties derived from numerical models. Hereby we consider noise that share the same linear univariate properties as real EEG recordings, namely the power spectra as well as the amplitude distribution in the time domain. Such data are represented by Iterated Amplitude Adjusted Fourier Transform surrogates (IAAFT; Schreiber and Schmitz, 2000). As a second example, time series are generated from so called Nf-Tori, which is a kind of a linear superposition of harmonic oscillations (Müller et al., 2005, 2006).

We find that the overall strength of cross-correlations within the functional network depends crucially on the pre-processing procedure, where higher frequency bands, which are usually associated with the performance of cognitive tasks (Kucewicz et al., 2014; Lachaux et al., 2012), are particularly affected. It may occur that the true spatial correlation structure is overshadowed by an excess of spurious correlations, simulating a kind of hypersynchronized dynamics. This is particularly true for highly autocorrelated EEG recordings, viz. a dominant contribution of slow frequencies, like those during deep sleep, post-seizure period of a focal onset seizure, subjects in a vegetative state or coma, beyond other physiological brain states. Even worse, the pre-processing process can cause correlations between data channels that can no longer be corrected by comparison with surrogate data, such that those artifacts induced in the functional network can no longer be distinguished from genuine cross-correlations. We show that this effect occurs not only in artificial situations when data are generated from numerical toy models, but also when real EEG data are used.

We provide a consistent explanation of the observed effect, which is sustained by numerical evidence and show how to eliminate such artifacts completely when using the appropriate pre-processing scheme.

## 2 Materials and methods

### 2.1 EEG acquisitions

#### 2.1.1 Sleep data

EEG sleep recordings were collected from 10 right-handed neurologically healthy male subjects recorded at the Sleep Laboratory of the Faculty of Psychology of the National Autonomous University of Mexico. The mean age ± SD of the group was 25.19 ± 3.19 years ranging from 21 to 31 years.

All subjects underwent a clinical interview before the study and maintained a 15-day sleep log. Only the subjects with regular sleeping habits and no symptoms of sleep disorders, history of medical, psychiatric, or neurological disorders, drug or medication were included. The participants gave their written informed consent and the experimental protocol was approved by the Ethical Committee of the Faculty of Medicine of the National Autonomous University of Mexico and followed the ethical standards of the Declaration of Helsinki (World Medical Association, 2024). The subjects slept two nights at the laboratory, the first night served for adaptation to recording procedures. The second night recording was used for scientific purposes.

EEG data were recorded using a Grass 8 to 20 polygraph with filters set at 0.1 and 70 Hz with a sampling rate of 1,024 Hz. According to the 10–20 International System (Homan et al., 1987) we used 19 electrodes (Fp1, Fp2, F3, F4, F7, F8, C3, C4, T3, T4, T5, T6, P3, P4, O1, O2, Fz, Cz, and Pz) referenced to A1. In addition, 2 electrodes were placed under the left and right clavicles to record the electrocardiogram (ECG), and 2 electrodes were placed below each eye to record the electro-oculogram (EOG), with filters at 10 and 70 Hz for EMG, and 0.3 and 70 Hz for EOG. All night standard polysomnography (PSG) data were digitized and stored using a 12 bit A–D converter of the GRASS-GAMMA acquisition program for offline analysis. Data segments with contamination with artifacts were identified by an expert (MC-C). The qualitative criteria includes a notable elevation of the EMG-signal, indicating strong ocular artifact. Furthermore, clear contamination of muscle activity as well as the saturation of the EEG-signal motivated the elimination of the corresponding segment. Removal was done simultaneously for all signals, such that the number of data channels remains always equal to 19.

Wakefulness and sleep stages were identified through standard procedures using 30-s epochs (Berry et al., 2012; Rechtschaffen and Kales, 1968).

Table 1 includes the percentages of time spent in unambiguously identified awake states or specific sleep stages. The percentages were calculated based on the total recording time for the entire night and generally correspond to the values expected for young adults (Swick, 2011; Williams et al., 1974). Before pre-processing, EEG was visually inspected and channels with increased impedance and permanent artifacts were discarded from further analysis. A 4th-order Butterworth low-pass filter (Butterworth, 1930) was applied to the EEG signals with a cut-off frequency of 45 Hz. Filtering was performed in both time directions (forward and backward) such that no phase shifts are produced via the filtering process. The filtered EEG was down-sampled to 128 Hz.

**Table 1:** Information about the 10 male subjects of the sleep recordings.

Subject	Age	% Wake	% NREM 1	% NREM 2	% NREM 3	% NREM 4	% REM
1	21	0.9	2.4	51.9	4.2	15.8	24.9
2	25	9.2	4.7	45.0	6.6	14.2	20.3
3	27	5.0	1.6	47.4	5.0	16.9	24.2
4	21	5.8	5.2	50.9	6.0	8.6	23.5
5	24	2.9	2.5	49.1	6.8	16.9	21.9
6	29	2.4	3.3	53.4	4.9	15.8	20.2
7	24	0.7	2.7	52.5	6.5	17.5	20.1
8	26	0.6	3.5	61.5	4.5	9.0	20.9
9	31	6.0	2.8	38.9	9.0	24.0	19.2
10	24	7.0	3.9	43.6	8.9	22.1	14.5

The Table includes the subject's age and the percentage of time spent in unambiguously identified awake states or specific sleep stages during the whole night sleep.

#### 2.1.2 Magnetoencephalographic recording

Here we use MEG recordings provided by the Consortium Human Connectome Project. A detailed description of the data can be found in Marcus et al. (2013) and Marín–García et al. (2024). In brief, we use approximately 3-h recordings on a whole head MAGNES3600 (4DNeuroimaging, San Diego, CA) at the Saint Louis University medical campus, resampled to a recording frequency of 508.63 Hz. From 248 MEG channels we use solely 126 that are common to all subjects. Five different test conditions were carried out: Resting state, two working memory tasks (0-back and 2-back) and two motor tasks (movement of either the right hand, left hand, right foot, or left foot). The dataset contains the recordings of 48 clinically healthy subjects.

#### 2.1.3 EEG pre-processing

We used two different pre-processing schemes to test their potential effect on the multivariate properties of the functional network:

- Filtering & Re-referencing (FR): EEG recordings were filtered in established EEG frequency bands. For each time point, the data sets were then referenced to the median of all electrodes.
- Re-referencing & Filtering (RF): The EEG data were first transformed to the median reference; thereafter, the signals were filtered in the traditional EEG frequency bands.

The band-pass filtering was performed by using a fourth-order Butterworth filter using the following definitions of the band borders (Schomer and Da Silva, 2012): δ-band 1.0–3.5 Hz, θ-band 3.5–7.5 Hz, α-band 7.5–12.5 Hz, β-band 12.5–25 Hz, γ-band 25–40 Hz. Broadband (Ω) was defined within the range of 1.0–40Hz and Ψ-band 0.1–40 Hz.

### 2.2 Numerical models

Here we focus on numerical models that allow for the control of the power spectra of the multivariate data set as well as the magnitude and spatial distribution of genuine cross-correlations.

#### 2.2.1 IAAFT surrogate data

As a first testing ground, we consider univariate Iterative Amplitude-Adjusted Fourier Transform surrogates (IAAFT) generated from the original EEG recordings (Lancaster et al., 2018; Schreiber and Schmitz, 2000). This method generates surrogate data that preserves the amplitude distribution in the time domain and the power spectrum of the original data, while Fourier phases are substituted by independent random numbers uniformly distributed within [0, 2π]. Due to the Wiener-Khinchin theorem, it is clear that by replacing the Fourier phases with random numbers, any signature of determinism and possible cross-correlations are destroyed (Aguilar-Hernández et al., 2024), so that the resulting time series are independent noise with the same linear univariate properties as the original recordings, but lacking any kind of genuine interrelations between the data channels (Lancaster et al., 2018; Schreiber and Schmitz, 2000). To generate the surrogate data, we focus on the sleep EEG of healthy subjects separating between different sleep stages.

Eventually we also used multivariate IAAFT surrogate data (Lancaster et al., 2018; Schreiber and Schmitz, 2000), where Fourier phases are replaced by random numbers as described above, such that Fourier phase differences between data channels are conserved. Therefore, the surrogate data maintain the linear univariate properties as well as linear cross-correlations of the original data.

#### 2.2.2 Nf-Tori

As a second model, we employ the so-called Nf-Tori (Müller et al., 2005, 2006), which is essentially the sum of numerous harmonic oscillations. Within this model, both the power spectra and the cross-correlation structure of the multivariate data set can be controlled. The corresponding model equation is given by:


$$\begin{array}{c} x_{l}\left(t\right)=\overset{N_{f}}{\underset{k=1}{\sum }}A_{lk}\sin\;\left(2\pi f_{k}t+\delta_{lk}\right) \end{array}$$ (1)

Here, *l* enumerates the time series whose maximum number is set to *N* = 19 to imitate the standard EEG using the international 10–20 system. The time unit is arbitrarily chosen as 128 data points, which coincides with the recording frequency of typical EEG recordings, and we consider *N*_*f*_ = 2, 000 sine waves. With the definition of a time unit one can define the frequency range of the signals. In the present case we consider frequencies between 1 and 40 Hz.

The distribution of the partial phases δ_*lk*_ (Müller et al., 2005, 2006) control interrelations between the different data channels. Because we aim to consider signals with zero genuine cross-correlations the δ_*lk*_ are chosen as independent random numbers that obey a uniform distribution within [0, 2π] (Aguilar-Hernández et al., 2024; Hramov et al., 2005).

Here we like to simulate colored noise, whose power spectral density follows a power law, that is, *P*(*f*) ~ *f*^−β^. Thus, we adjust the coefficients to $A_{lk}=f_{k}^{-\frac{\beta }{2}}$ with β = [0, 1, 2, 3] to test whether the characteristics of the power spectra can influence the estimation of the linear cross-correlations when using the two pre-processing schemes.

### 2.3 Statistical treatment

#### 2.3.1 Global correlation

A multivariate data set was segmented into 30-s time windows following sleep stage classification rules (Rechtschaffen and Kales, 1968). The zero-lag cross-correlation matrix denoted as *C*_*ij*_(*t*), was calculated for every non-overlapping time window. We define the global correlation per time window at instant *t* as the mean of the non-diagonal elements of the corresponding correlation matrix *C*_*ij*_(*t*):


$$\begin{array}{c} gc\left(t\right)=\frac{2}{N\left(N-1\right)}\overset{N}{\underset{i>j}{\sum }}C_{ij}\left(t\right) \end{array}$$ (2)

#### 2.3.2 Estimation of genuine cross-correlations

Random cross-correlations, viz. non-zero cross-correlation estimates of otherwise uncorrelated signals, appear because estimates are undertaken using finite data segments. These coefficients are symmetrically distributed around zero (positive and negative coefficients appear with the same probability). The width of the distribution depends on the length of the data segment as well as the spectral content, viz. the power spectral density of the signals (Müller et al., 2011).

In addition to the concepts derived from random matrix theory (Laloux et al., 1999; Plerou et al., 1999, 2002; Müller et al., 2005, 2006), genuine cross-correlations can be separated from random contributions via the comparison with surrogate data that have the same linear univariate properties as the original data, but no signature of determinism and no correlation between the data channels. In Marín-García et al. (2013) the performance of several surrogate based measures have been evaluated via the comparison with analytical results. The performance of several surrogate-based measures has been evaluated by comparison with analytical results. It turned out that the so-called “Significant Average Correlation” matrix (SAC) provides the most accurate estimates. In addition, this matrix takes the multivariate character of the EEG measurements into account. Instead of condensing all the information into a single scalar quantity (Carmeli et al., 2005; Cui et al., 2010; Müller et al., 2011), a type of a linear interrelation matrix is estimated. For each data window and for each pair of data channels a quantitative comparison with IAAFT surrogates is realized, using a well-defined, Bonferroni corrected significance level. Given the symmetrical probability density of random correlations, their amount can be essentially reduced via some average process. Thus, the elements of the resulting SAC-matrix reflect almost precisely genuine correlations. In this paper, we use this technique to evaluate the performance of the two pre-processing schemes under consideration.

#### 2.3.3 Time duration an electrode serves as median reference

We applied both pre-processing schemes (FR and RF) to real EEG recordings and the data derived from both models (NF-Tori and IAAFT surrogates). The original reference for the EEG recording was the left earlobe A1. Subsequently, the data were re-referenced to the median. The median at a fixed time taken over an odd number of EEG channels is given by the electrical potential measured by a certain EEG channel, which is taken as the reference signal. Is to say, this signal is subtracted from each data channel of the EEG, such that at each time point at least one signal, that one chosen as the reference signal, is precisely zero.

We define the duration Δ*T* for which an electrode serves as an EEG reference as the consecutive number of sampling points at which its electrical potential assumes the central value of the entire multivariate recording. Finally, we represent the probability distribution of these time spans as a rank-frequency distribution.

#### 2.3.4 Spectral slope

We calculated the average power spectrum using a non-overlapping running window length of *T* = 30 s. The slope was then estimated separately for each sleep stage, assuming that the spectral power follows approximately a power law.


$$\begin{array}{c} PSD\left(f\right)\sim \frac{1}{f^{\beta }} \end{array}$$ (3)

## 3 Results

### 3.1 Comparison of the correlation structure derived for MEG and EEG in different reference schemes

Figure 1a shows a cross-correlation matrix derived from MEG recordings of a person performing a motor task. The matrix displays an average over all trials. In addition, the number of MEG detectors was reduced to 19 by selecting the signals of those detectors whose locations are closest to those of the international 10–20 system. Other reduction methods lead to quantitatively almost identical average matrices (Marín–García et al., 2024). On the other hand, Figures 1b, c show results derived from EEG recordings of a healthy young male during REM-sleep and the awake state during rest. One easily appreciates the outstanding structural similarity between the three matrices. This similarity between MEG and EEG recordings was quantitatively confirmed for 48 subjects elaborating two motor tasks, two working memory tasks, and the rest condition (Marín–García et al., 2024). The similarity between the cross-correlation matrices averaged over all conditions separately for the different subjects, estimated by the Pearson coefficient, is about 0.75. Figure 1d shows an example of a cross-correlation matrix derived from MEG-recordings of a subject averaged over all trials of the five different conditions mentioned above. Finally, Figure 1e depicts the functional network represented by an average cross-correlation matrix corresponding to sleep stage 2 using the median reference, while Figure 1f depicts the same using the earlobe A1 as a reference point.

**Figure 1:**
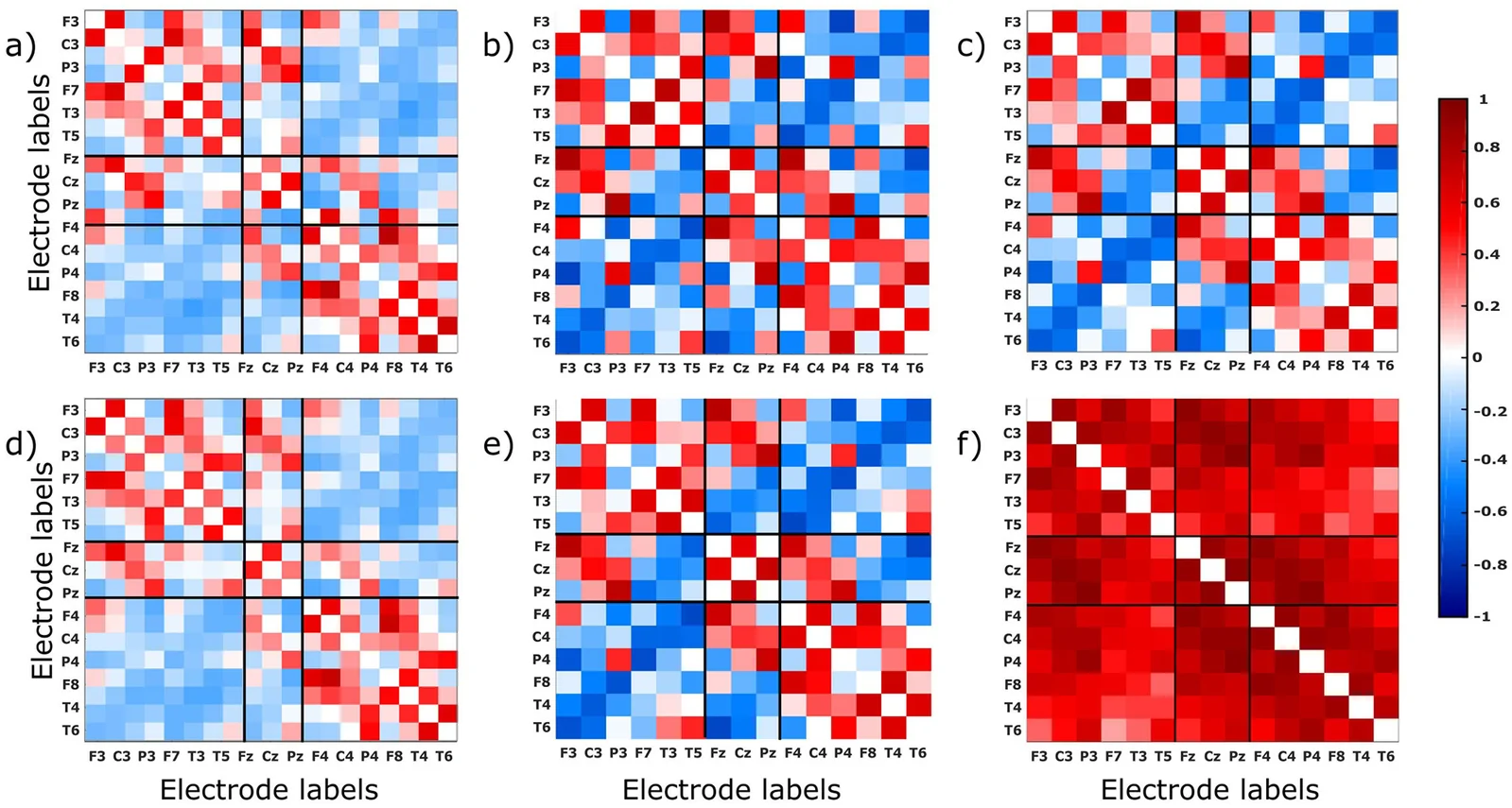
Average correlation matrices extracted from MEG and EEG recordings. **(a)** Displays the average cross-correlation matrix estimated from MEG data during the performance of a motor task. **(b, c)** Show the correlation matrices from EEG data with the median as a reference scheme of a healthy young male during REM-sleep and rest conditions. **(d)** Shows the average correlation matrix derived from MEG data considering all trials of 5 different conditions. **(e)** Shows the average correlation matrix of EEG signals estimated over the episodes of light sleep (sleep stage 2) of a healthy young male using the median reference, and **(f)** shows the same using the left earlobe A1 as a reference point. To make the correlation matrices, estimated for MEG and EEG recordings, comparable, 19 MEG detectors with the closest locations to the international 10–20 system were selected.

The similarity between the average matrices derived from the MEG-recordings with that obtained for the EEG using the median reference is evident, which suggests that the median reference is the most suitable reference when constructing the functional network. This result has been obtained quantitatively in the past, both using numerical modeling to generate synthetic data under controlled conditions (Rios et al., 2019) and using real MEG and EEG recordings (Marín–García et al., 2024). We will therefore concentrate on this reference scheme in the sequel.

### 3.2 Influence of the pre-processing scheme on the correlation analysis

To test whether the pre-processing schemes (FR) and (RF) influence the numerical estimates of the cross-correlations, we consider the global correlation (Equation 2) of an EEG recorded from subject 8 during night sleep as a crude measure. Figure 2 displays the results. The Figure 2a shows the results obtained using the RF scheme and the Figure 2b the corresponding outcome for the FR scheme. *gc*-estimates are shown for each frequency band in a color-coded version. The corresponding hypnogram is provided at the bottom of each panel.

**Figure 2:**
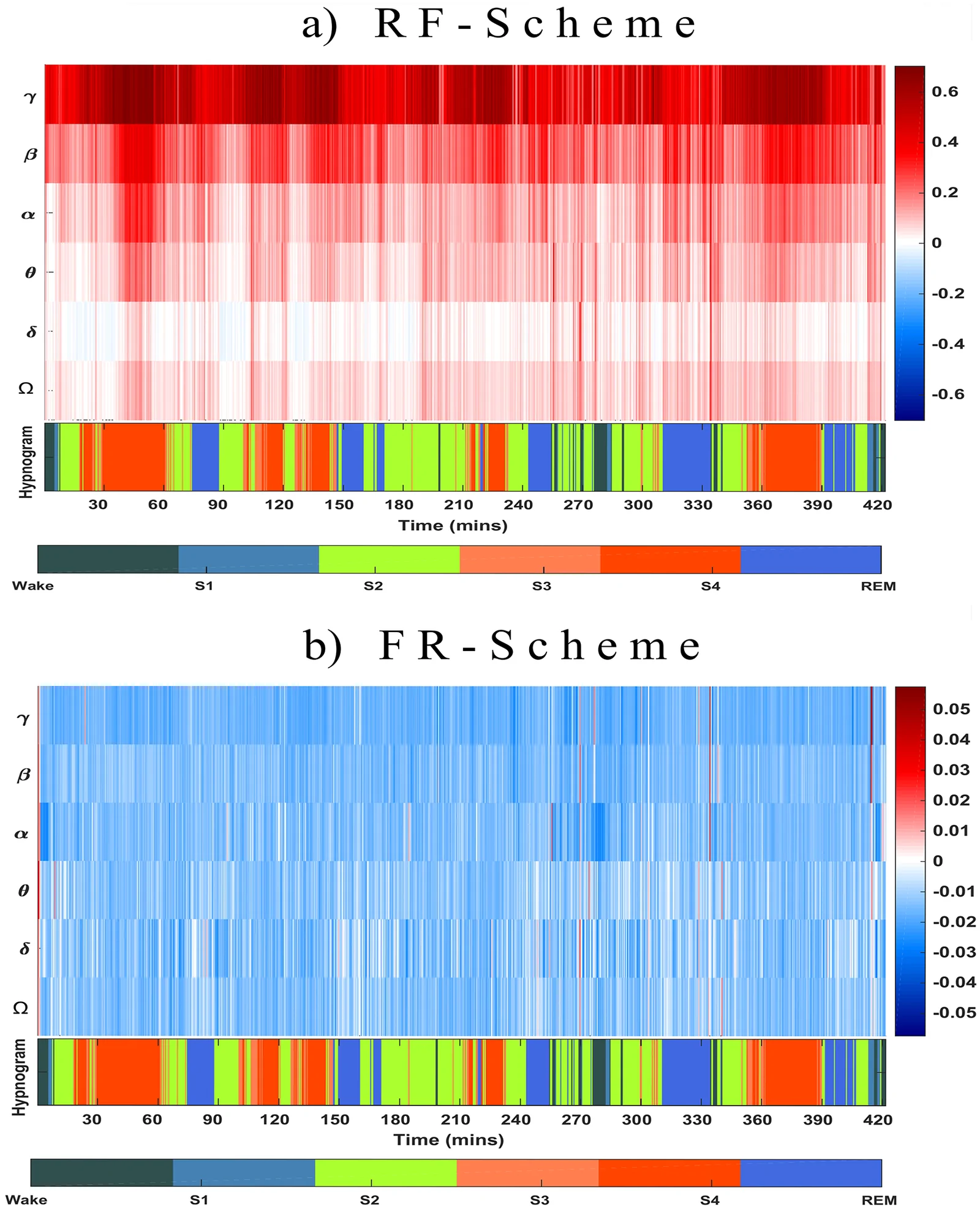
Time evolution of the global correlation. *gc*(*t*) for subject 8 is shown using a 30 s running window, which is shifted without overlapping over the whole night recording. The vertical axis indicates results obtained for different frequency bands. At the bottom of each panel the color-coded hypnogram is displayed (Wake = dark green, S1 = dark blue, S2 = green, deep sleep S3 and S4 = salmon and red, and REM = blue). The upper panel shows the estimates for the RF scheme and the lower panel those for the FR scheme. **(a)** RF scheme. **(b)** FR scheme.

The difference between the two procedures is evident. When using the RF scheme, we observe a predominance of positive correlations, which is more pronounced for the higher frequency bands. In contrast, for the FR scheme (Figure 2b), only slightly negative global correlations are observed with no apparent alterations in the different frequency bands. This behavior is intuitively plausible by recalling the correlation pattern shown in Figures 1a–e, where positive and negative entries appear almost in the same proportion.

Figure 3 displays box plots of the distribution of the non-diagonal elements collected for all subjects of a set of cross-correlation matrices generated by a 30-s non-overlapping running window. Different panels show the results separately for different sleep stages as well as for different frequency bands. The upper and lower panels show the results obtained for RF and FR schemes, respectively. The qualitative difference in the distributions is again evident. One observes that all results obtained for the RF-scheme are shifted toward positive values, while estimates for the FR procedure are slightly negative on average. In addition, a strong increase in the mean values for higher frequency bands can be observed when using the RF-scheme, while at the same time, the distributions become narrower. This is particularly true for sleep stage 4, simulating a hypersynchronized dynamical state. For the FR-scheme, on the other hand, the mean values remain approximately constant across frequency bands; solely the widths of the distributions tend to decrease for the β and γ-band.

**Figure 3:**
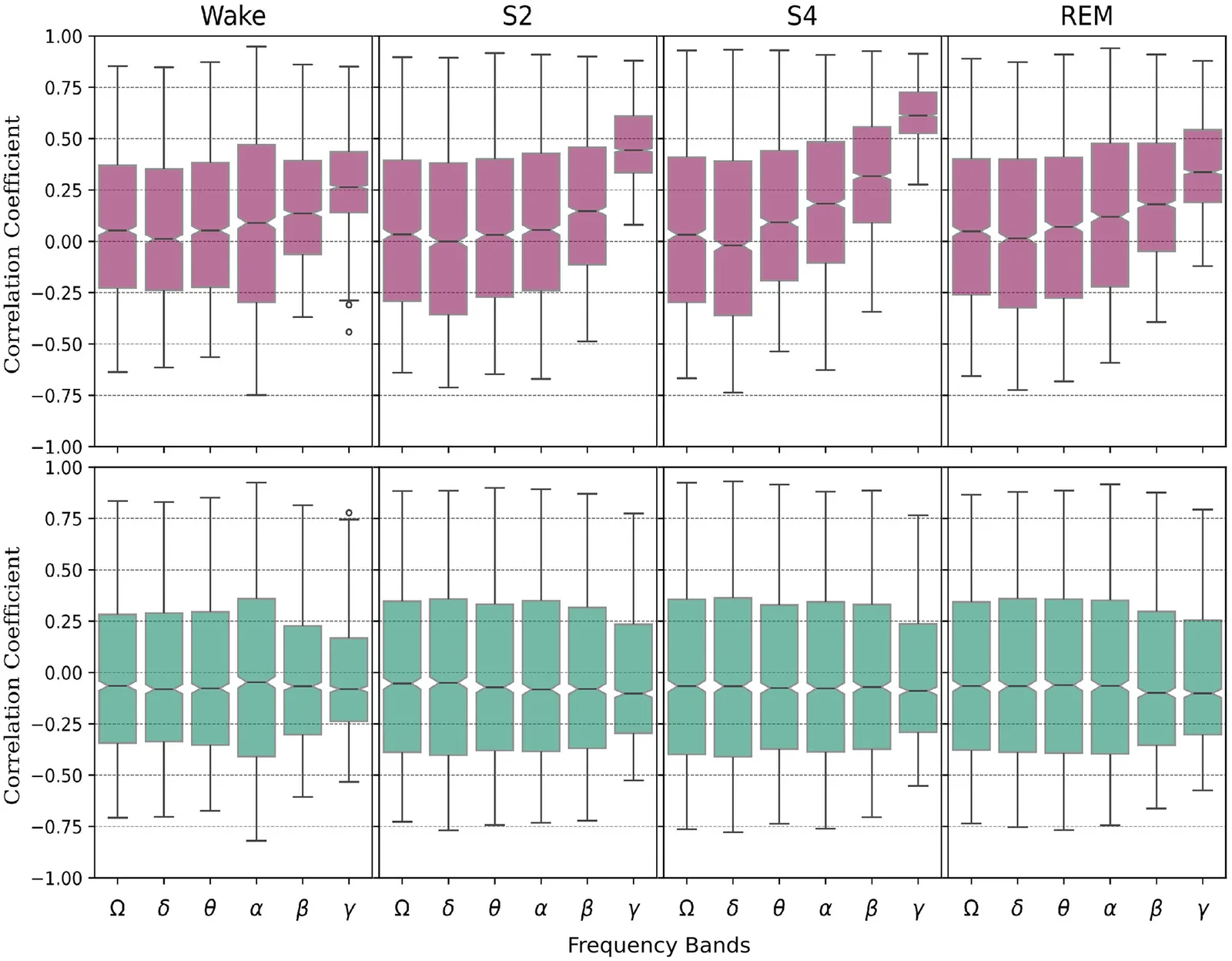
Distribution of cross-correlation coefficients separately for different sleep stages and frequency bands. Data were collected for all 10 subjects with a 30 s non-overlapping running window. Top/bottom row display results obtained for the RF/FR pre-processing scheme, respectively.

### 3.3 Univariate IAAFT data and Nf-Tori model

The univariate IAAFT surrogates, generated from the sleep EEG recordings, preserve the power spectrum as well as the amplitude distribution in the time domain while all genuine cross-correlations get eliminated. Thus, only random correlations, provoked by finite size segments and the shape of the power spectra (Müller et al., 2011) remain as sources for the production of random correlations and it is to be expected that for such data the non-diagonal elements of the correlation matrix are closely distributed around zero, which explains the much narrower distributions shown in Figure 3.

However, contrary to this expectation, we observed a pronounced increase of positive cross-correlations when data were re-referenced to the median *before* band-pass filtering. This is particularly true for the β and γ-band and is even more pronounced for surrogates generated from data segments that belong to sleep stages 2 and 4. Instead, for the FR-scheme, median values of the distributions of cross-correlation coefficients are systematically located at slight negative values around −0.025, as documented in Figure 4, without any significant change across frequency bands. Figure 4 displays the results obtained for univariate IAAFT-surrogates, where all genuine cross-correlations have been destroyed. Thus, one should expect that the distribution of non-diagonal elements should be centered at zero. However, the same RF-pre-processing has been applied to these data sets. The effect of the pre-processing scheme is evident.

**Figure 4:**
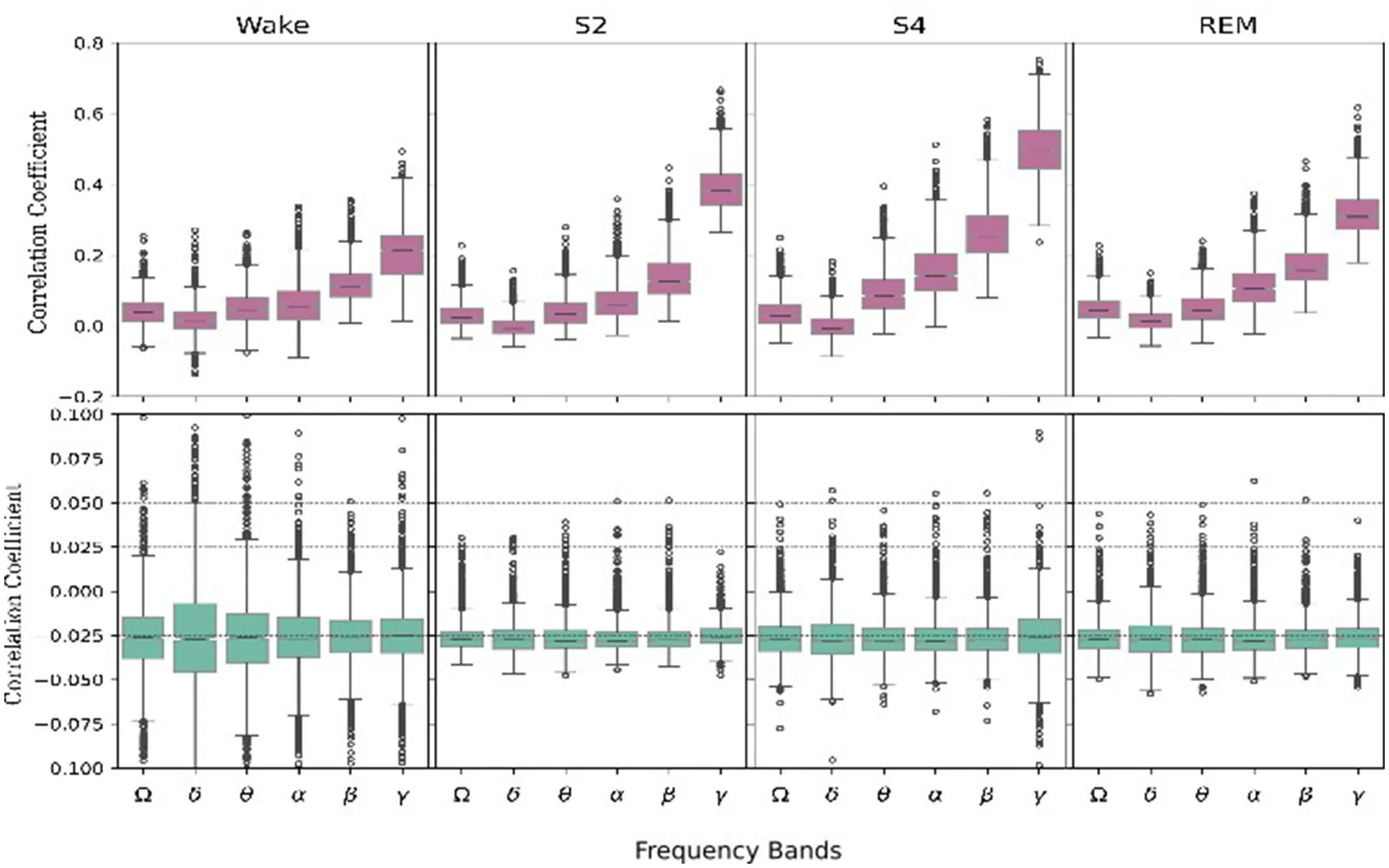
Results obtained for univariate IAAFT-surrogates generated for different sleep stages. Box plots represent the distribution of non-diagonal elements of correlation matrices averaged separately for different sleep stages and frequency bands. The top and bottom panel display the results obtained using the RF and FR pre-processing schemes, respectively. For each 30 s segment, we produced one IAAFT surrogate.

This result demonstrates that for both the FR and the RF-scheme, spurious effects on the cross-correlation coefficient are induced. However, in addition to the fact that the effect size is significantly larger in the case of the RF scheme, we also observed a strong dependence on the physiological state, i.e., on the different sleep stages. As we find that the artificial increase in positive cross-correlations in univariate IAAFTs is most pronounced during deep sleep, which is characterized by high-amplitude slow frequency oscillations, it suggests that these results are provoked by the different spectral composition of the signals during e.g., deep sleep and awake state.

To provide further evidence of the influence of the power spectra we next turn to the Nf-Tori to produce signals with different spectral content but zero genuine cross-correlations. To this end, we adjust the amplitudes *A*_*lk*_ of Equation 1 such that signals show power law spectra ~*f*^−β^ with β = 0 (white noise), β = 1 (pink noise), β = 2 (red noise), and β = 3 (Equation 3). The different columns of Figure 5 display the corresponding results.

**Figure 5:**
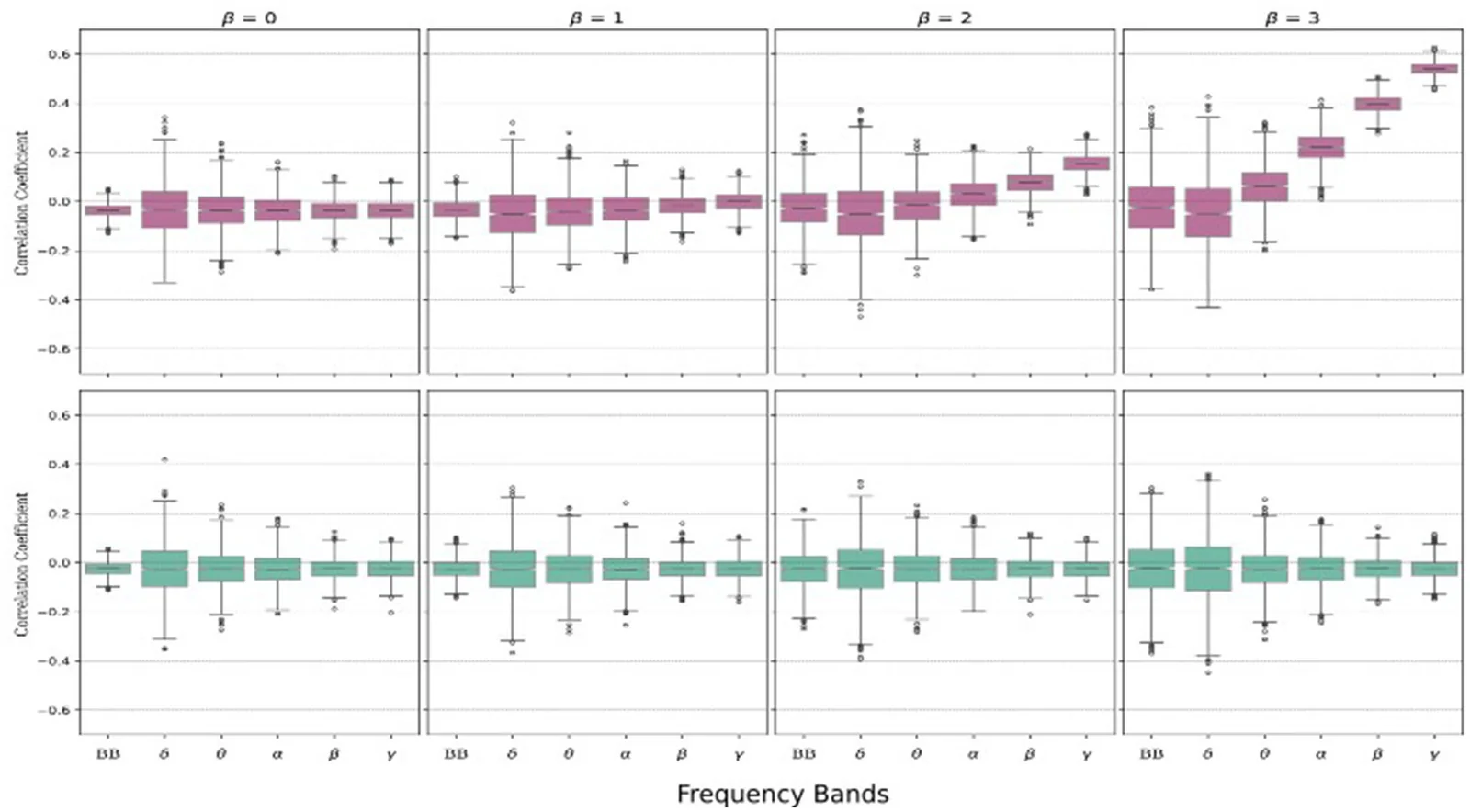
Results for the Nf-Tori simulating zero genuine cross-correlations and different power spectra. Box plots represent the distributions of the cross-correlation matrix's non-diagonal elements for different power spectral slopes (β = 0, 1, 2 and β = 3). The upper and lower panels correspond to the RF and FR pre-processing schemes, respectively. For each β-value we produced 10 data segments of 3,840 data points each, which corresponds to a 30 s EEG segment with a recording frequency of 128 Hz.

The top row shows the results for the RF-scheme. As in the case of the IAAFT surrogates, we observe an increase in the center of the distributions accompanied by a decrease in their widths, which is more pronounced the steeper the frequency dependence of the power spectra. At this place it is worth noting that β-values between 2 and 3 are within the range of the real empirical spectral slope of sleep data as documented e.g., in Figure 6 below. In this Figure it can be observed that wake, light sleep in stage 1 (S1), and REM-sleep exhibit larger medians for the spectral slope ([−2.5, −2.0]). On the other hand, deep sleep stages S3 and S4 have the largest slope values (β~3), which aligns with the physiological knowledge of the spectral features (Pereda et al., 1998). The slopes obtained for sleep stage 2 (S2) are located between those of deep and light sleep, with slopes close to −2.5. Note, that similar results could be observed for other EEG recordings like, e.g., the post-ictal period of focal onset seizures (Müller et al., 2011; Voytek et al., 2015), newborns (Schaworonkow and Voytek, 2021) and young children during wake state (Arnett et al., 2022), as well as for patients in vegetative state or coma (Maschke et al., 2023).

**Figure 6:**
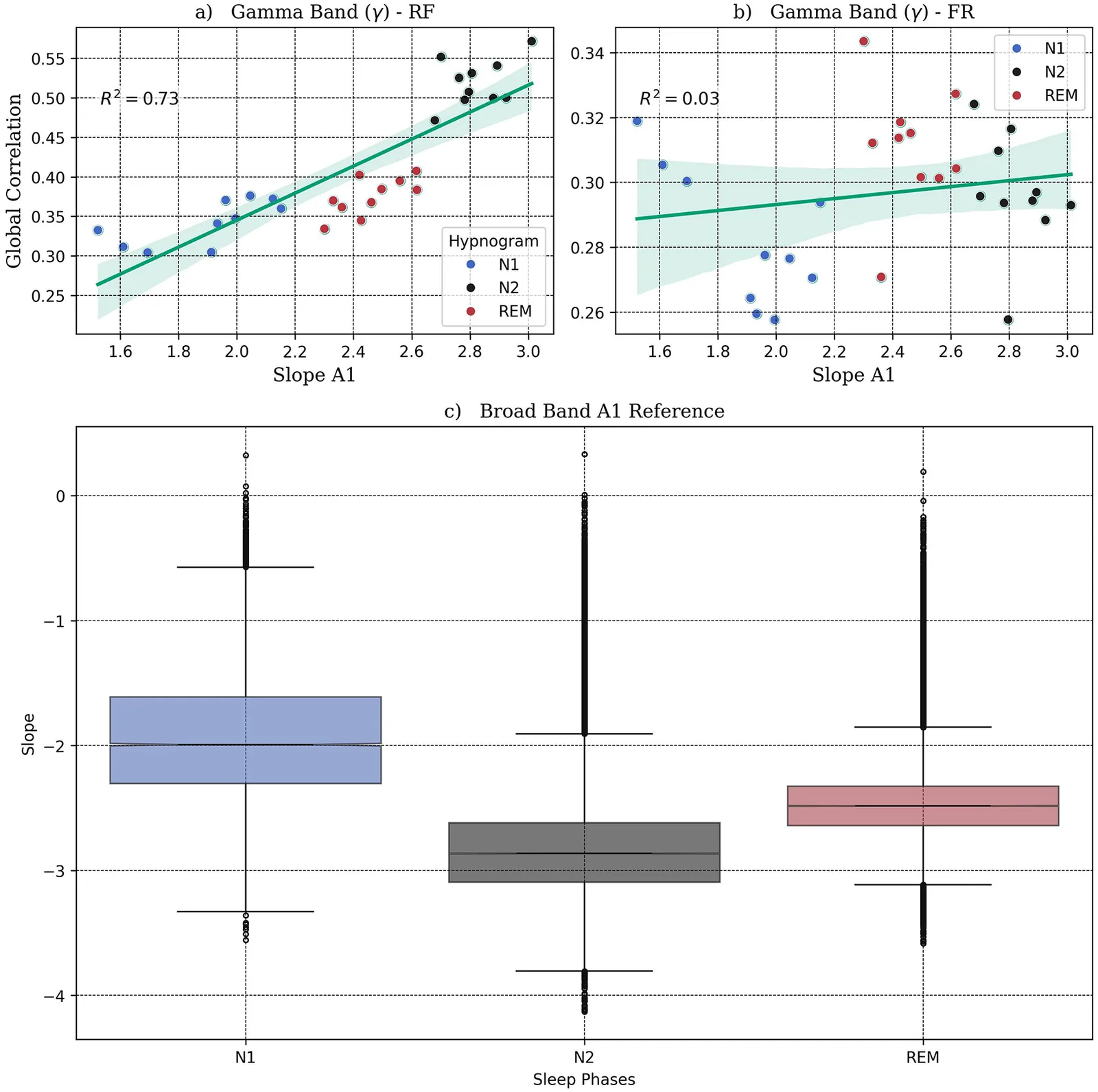
Power spectral slopes and global correlation. The figure depicts the subject averaged values of the spectral slope vs. global correlation in the γ-band for RF- and FR-schemes in **(a, b)**, respectively. In this case, global correlations are estimated using the absolute values of the Pearson coefficients. Blue dots represent light sleep stages N1 (S1 and S2), black dots correspond to N2 (deep sleep) and red dots to REM sleep. **(c)** Shows the spectral slope for EEG data using the earlobe reference. Each box-plot shows the distribution of the slope values β of the power spectra separately for each sleep stage for the 10 EEG recordings measured with the earlobe reference.

These results correlate with those shown in Figure 5, the more pronounced the slope of the power spectral densities, the larger is the amount of artificially induced cross-correlations. In the same spirit, these findings fit nicely with those shown in Figure 2, which illustrates a maximum increase in correlations for deep sleep stages that have steeper spectral slopes. Results obtained for the FR-scheme, on the other hand, do not show any dependency on the spectral slope. Distributions are again slightly but systematically shifted toward negative values.

In this section, we provided clear evidence that the increase in cross-correlations for higher frequency bands using the RF-scheme is strongly correlated with the power spectra of the signals, viz. the spectral slope. Next, we try to uncover the mechanism by which the shape of the power spectra provokes this undesired spurious effect.

### 3.4 Duration an electrode serves as the median reference

Figures 7a–c displays the rank order distribution of the duration for which a data channel serves as a reference (as described in Section 2.3.3). Here we show results for the Nf-Tori as well as for EEG data for subjects during whole night sleep. The diagrams display the frequency with which a certain duration occurred in units of sampling points.

**Figure 7:**
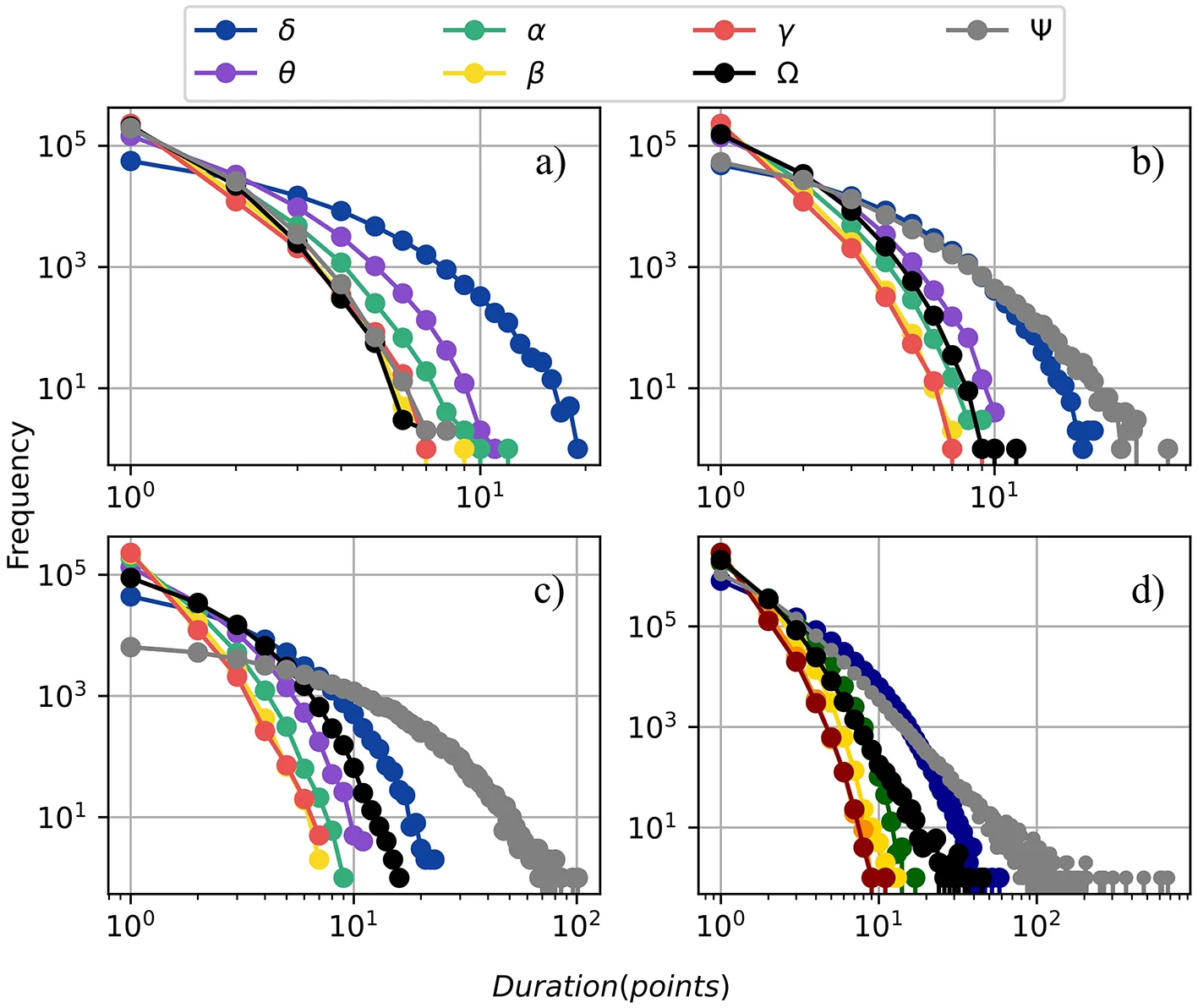
Rank-order distribution of the duration in sampling points for which a particular data channel serves as the median reference. **(a–c)** Display results obtained for the Nf-Tori model with different slopes of the power spectra: **(a)** β = 1, **(b)** β = 2, and **(c)** β = 3. **(d)** Shows the results obtained for the whole night sleep including all sleep stages using the earlobe reference A1. Results for different frequency bands are drawn in different colors: δ (blue), θ (purple), α (green), β (yellow), γ (orange), Ω (black 1 − 40 *Hz*), and Ψ (Gray 0.1 − 40 *Hz*).

For β ≤ 1 differences between the distributions are tiny and the longest durations are all below 20 data points. In this case, there is almost no discrepancy if re-referencing is performed before or after filtering, but differences get more pronounced for steeper spectral slopes. For increasing β-values, the probability for the occurrence of longer durations increases successively for the broad-band cases (Ω and Ψ-band). Figures 7a–c display results obtained for the Nf-Tori model with different slopes of the power spectra: (a) β = 1, (b) β = 2, and (c) β = 3. Figure 7d shows the results obtained for the whole night sleep including all sleep stages. Results for different frequency bands are drawn in different colors: δ (blue), θ (purple), α (green), β (yellow), γ (orange), Ω (black 1–40 Hz), and Ψ (Gray 0.1–40 Hz). It can be seen that the gray curve shows the longest tail toward larger durations in comparison to all other frequency bands, at least for β≥ 2. Maximum values are up to hundreds of recording points. Given that the Ω and Ψ-band cover almost the same frequency range, this result is remarkable at first glance. The only difference to the Ω-band is that the Ψ-band includes slow frequencies between 0.1 and 1 Hz, which in addition enter with larger amplitudes the steeper the slope of the power spectra. This result underlines that high amplitude and slow frequency components are the origin of the notable increase of spurious cross-correlations in high frequency bands.

This effect contrasts with the distributions of fast frequency bands such as the β or γ-band, which has a huge number of short durations and suppresses events longer than say 10 recording points. The duration of an electrode serving as the reference depends strongly on the frequency content of the signals.

Thus, if re-referencing is done *before* filtering, slow frequency large amplitude components of the broadband signal may dominate. Then the slowest dominant frequency component determines the shape of the corresponding distributions, i.e., the slower the dominant frequencies, the greater the probability for the occurrence of long durations. This leads in turn to redundant information induced over longer periods of time in all data channels and thus to an artificial increase in cross-correlations. This discrepancy between (i) the duration a data channel acts as the reference and (ii) a typical time scale of a band-passed filtered signal is highest for fast frequency bands. Therefore, the effect is most pronounced for the β and γ-band as reported above. This situation is presented transparently in the next subsection.

We like to emphasize that the impact of the present study is by far not restricted to sleep research. Drastic changes in the spectral composition of EEG recordings are also observed, e.g., during the peri-ictal transition of focal onset seizures, with a dominant contribution of slow frequency components (and the absence of higher frequency power) at the final part of an epileptic seizure as well as during the immediate post-seizure period.

Exemplary, we show that the results obtained for the Nf-Tori are also relevant for real world data, like the sleep recordings considered here. As documented in Figure 7d, the distribution of durations extracted from the EEG signals measured in the earlobe reference A1 during sleep, is highly similar to the results obtained for the Nf-tori with β = 3. Thus, similar effects have to be expected.

### 3.5 Spectral slope vs. global correlation

We estimated the exponent β of the spectral slope of the power spectrum from the sleep data of the 10 EEG signals recorded with the A1 reference. Each sleep stage was analyzed separately, using a non-overlapping 30 s running window, and values were averaged per subject and sleep stage. Figure 6 displays the numerical estimates for the slope-value vs. global correlation in the γ-band for the RF and FR scheme in the top-left and top-right panels, respectively. Note, here global correlations are estimated using the absolute value of the Pearson coefficients, given that here we are interested in the comparison of the absolute amount of cross-correlations.

Blue dots represent light sleep stages S1 and S2, black dots correspond to deep sleep, and red dots to REM sleep (Berry et al., 2012). The figure reveals a positive correlation of roughly 0.85 and *r*^2^= 0.73 between the spectral slope and the global correlation in the γ-band for RF-scheme. For N2, global correlation encounters values above 0.5. In contrast, for the FR-scheme we obtained a correlation of 0.17, a *r*^2^ = 0.03 and *gc*-values are somewhat distributed around 0.3.

In the bottom panel of Figure 6 it can be observed that N1 and REM-sleep exhibit larger medians for the spectral slope ([−2.5, −2.0]). On the other hand, N2 have the lowest slope values (β ≃ −3), which is consistent with the physiological knowledge of spectral features in sleep. These results are consistent with those shown in Figure 2 for the RF-scheme, which illustrates a maximum increment in correlations for deep sleep stages (N2) with pronounced spectral slopes.

### 3.6 The global average, the median reference and the induction of genuine cross-correlations

The authors of Goldman (1950) proposed the usage of the global average reference, based on the assumption that the sources of electrical activity recorded with EEG originate from neural dipoles with random positions and orientations. Following this idea, the magnitude of the average reference should be close to zero. One limitation of the global average reference is that outliers influence it. Specifically, if one channel has a higher amplitude than the others, this event will affect the remaining signals when re-referencing the EEG series. Figure 8a shows this case using the Nf-Tori model lacking genuine cross-correlation. Here we select three channels with higher amplitude than the others.

**Figure 8:**
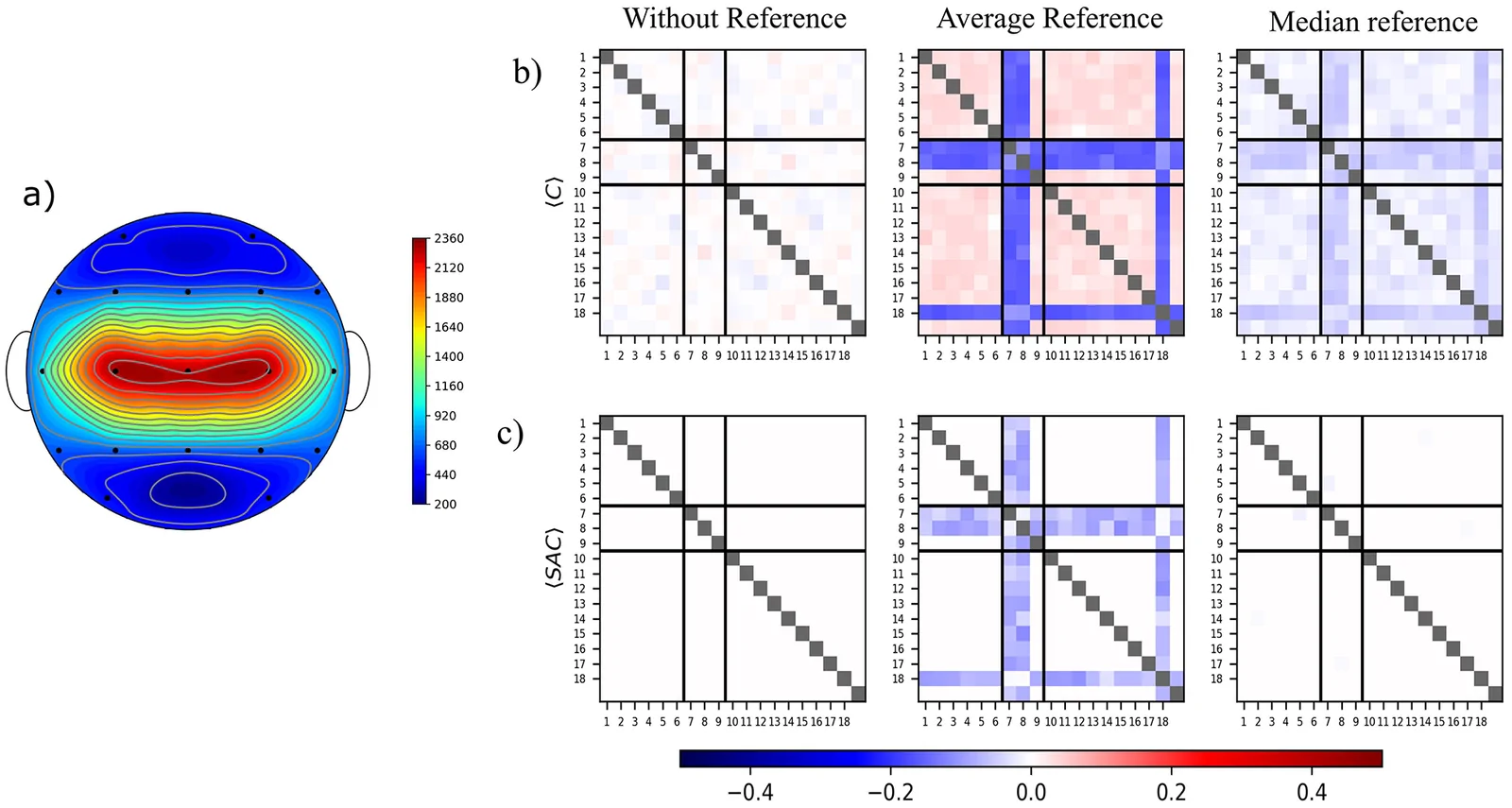
Influence of the signal amplitude: comparison between global average and median reference. **(a)** Shows a heat map of the color-coded amplitude of the signals derived from the Nf-Tori model. Independent signals (absence of genuine cross-correlation) are created but with three central electrodes exhibiting an amplitude 10 times higher than the remaining ones. Note, that may simulate the situation when a few data channels are contaminated by muscle artifact. **(b)** Presents the correlation matrices for original reference free data, those using the global average reference, and matrices using the median reference (from left to right). **(c)** Provides results for the same cases but using significant average correlation (SAC).

Figure 8b shows the correlation matrices for the original data, when re-referencing the signals to the global average and employing the median reference. We observe that the global average induces coupling (positive correlations) between signals with low amplitude. This is consistent with the observation that higher amplitude signals dominate the global average and thus induce redundant information in lower amplitude signals, which in turn translates into positive cross-correlations across these channels. In contrast, negative correlations are observed within the group of high amplitude signals, leading to a peculiar spatial correlation pattern. The median, on the other hand, is less influenced by amplitude differences and the resulting pattern is much less pronounced.

Note that the observed spatial cross-correlation pattern is not due to genuine interrelations between the data channels, but is solely provoked by differences of the amplitudes of the signals. Here, the question arises of whether one is able to repair this effect when estimating surrogate-corrected significant average correlations (Marín-García et al., 2013; see subsection SAC). Figure 8c presents the corresponding results.

For the original data derived from the Nf-tori, the corresponding matrix shows just zero entries, as it should be, because by construction the data channels are completely independent. The same applies when using the median reference. The undesired generation of linear interdependencies is almost completely corrected. However, when considering data referenced to its global average, also the SAC-matrix shows qualitatively the same intriguing pattern as when the usual cross-correlation matrix is estimated, although now the pattern is less pronounced. In any case, for this reference scheme, one would expect functional network distortions due to amplitude differences between signals, such as those caused, for example, by muscular or ocular artifacts. This result not only underlines the superior performance of the median reference, but additionally recommends the usage of the surrogate corrected version of the cross-correlation matrix (Marín-García et al., 2013).

### 3.7 Does the SAC-matrix correct for the negative effects caused by the pre-processing procedures?

Motivated by the results obtained in the former section, one may ask if the SAC-matrix is also able to correct the artifacts caused by the two pre-processing schemes reported above. To probe for its performance in such situations, we use data that do not contain any genuine cross-correlations (this is actually the null hypothesis of the cross-correlation analysis), but adopt all linear univariate properties of real EEGs, viz. we are using univariate IAAFT-surrogates. In order to consider a case that generates a considerable effect size we focus in this section exclusively on data segments of sleep stage 4 of all 10 subjects. We discuss the RF as well as the FR preprocessing scheme using the standard correlation matrix as well as the SAC-matrix. Results are displayed in Figure 9.

**Figure 9:**
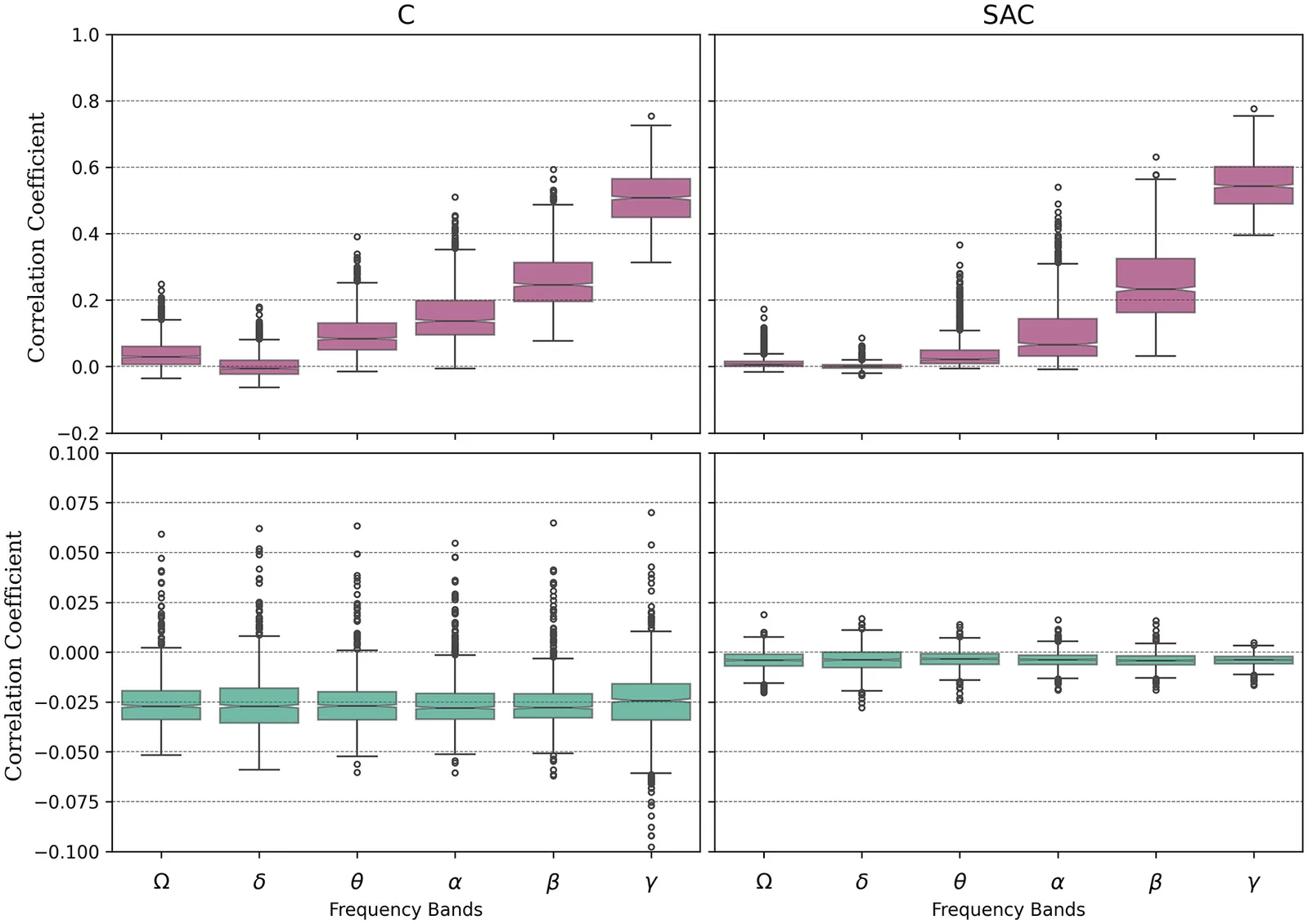
Results obtained for univariate IAAFT-surrogates generated for sleep stages 4 of all 10 subjects. The boxplots represent the distribution of non-diagonal elements of correlation matrices for sleep stages 4 obtained for univariate IAAFT-surrogate data. The left column shows results for the usual cross-correlation matrix, right column corresponding results for the SAC-matrix. The top and bottom panel display the outcomes using the RF and FR pre-processing schemes, respectively.

For both interrelation matrices the RF-scheme induces notable positive cross-correlations especially in higher frequency bands. For the γ-band the displacement is 0.5 on the average for the C-matrix and even larger for the SAC-matrix, with fluctuations that yielding estimates up to 0.75, thus considerable high values.

On the other hand, using the FR-scheme we observe for the C-matrix a small shift toward negative values of about −0.025, consistently for all frequency bands. The distributions are asymmetric for the α- to the β-band with large fluctuations toward positive values. Solely the distribution for the γ-band is more symmetric, fluctuating similarly in both directions. Thus, distortions are also caused for the FR-scheme, although they are much smaller than for the RF-pipeline.

The situation gets more favorable when considering the SAC-matrix. Now the displacement toward negative values gets essentially eliminated and also the widths of the distributions are much smaller. Only a tiny shift, smaller than −0.01 remains, which gets slightly more pronounced for higher frequency bands. However, distortions of this magnitude are tiny and get even more reduced if the spectral slope of the EEG-signals is less pronounced, as i.e., for REM-sleep. In fact, the effect is at least one order of magnitude smaller for the FR-scheme using the SAC-matrix in comparison to the RF-procedure. Thus, we may conclude that distortions caused by the FR-scheme are almost insignificant for real world applications, when surrogate corrected cross-correlations are considered. If, on the other hand, data are re-referenced before filtering, distortions may be huge and irreparable.

## 4 Discussion

Different neuroimaging techniques allow for the estimation of functional networks through recording measures such as EEG, MEG, functional magnetic resonance imaging (fMRI), Positron Emission Tomography (PET), etc. A central point for the construction of the functional network in EEG measurements is the reference scheme. In Rios et al. (2019), the usage of the median or average reference is proposed for the construction of the functional network because it causes the least amount of topological distortions. On the other hand, in Marín–García et al. (2024), it is shown that using the median reference in EEG recordings results in a functional network that topologically resembles the one obtained from reference free MEG records.

In this work, we implement the median reference for 10 EEG recordings from healthy subjects during sleep as a testing ground. We report the existence of a spurious effect in the estimation of the functional network connections that depends on the pre-processing procedure of the data. We observe a potential increase of the overall correlations with the frequency band.

Using data derived from numerical models as well as real world EEG data, we conclude that the functional network, constructed via linear cross-correlations, may strongly dependent on the way data is pre-processed, namely whether it is first re-referenced and then band-pass filtered, or vice versa.

This is particularly true for recordings with strong autocorrelations like those of sleep stages 3 and 4, where the spectral slopes encounter values even below −3. But, as documented, also results obtained for sleep stage 2 are notably affected. The excess of positive correlations overshadows the true spatial correlation structure during these sleep stages, simulating a kind of hypersynchronized dynamics, a situation that certainly leads to wrong conclusions.

Intuitively one may incline in favor of the RF-scheme, because of its reduced workload. In this case, re-referencing should only be done once, just before filtering. However, it turned out that in this case the reference signal may be strongly influenced by high amplitude slow frequency components, which can induce redundant information in all data channels and thus give rise to the occurrence of positive correlations covering the whole network. This effect manifests mainly in high frequency bands, where the discrepancy of the different time scales, the large time scale of the reference signal and the fast one of the high frequency range, is most pronounced. Moreover, this distortion is irreversible and cannot be repaired by, e.g., the estimation of surrogate corrected cross-correlations.

On the other hand, when re-referencing each of the band-pass filtered signals individually, distortions are notably reduced and, in this case, almost eliminated when the SAC-matrix is estimated. In this framework the pre-processing scheme solely causes tiny (negative) random correlations, which can be corrected with an appropriate usage of surrogate data.

Again, we would like to emphasize that in the present study, we used sleep recordings as an appropriate testing ground because of the pronounced changes in the spectral composition during sleep cycles. Thus, within the same recordings we could analyze data segments with notable changes in the spectral slope. However, we expect similar results for signals like: the post-ictal period of focal onset seizures (Müller et al., 2011; Voytek et al., 2015), newborns (Schaworonkow and Voytek, 2021), young children during the awake state (Arnett et al., 2022), patients in coma (Maschke et al., 2023), and insomnia (Corsi-Cabrera et al., 2012) beyond others. Actually, the power spectra of e.g., EEGs of young children show steep slopes around −3. Therefore, the results obtained in the present study are not restricted to sleep recordings but have a much broader impact.

As a conclusion, although demanding a higher computational cost, we recommend the FR-scheme as well as the usage of surrogate corrected cross-correlations as proposed by the SAC-matrix. According to the present study, distortions of the functional network are minimized in this case and even the small perturbations can be almost eliminated by the proposed scheme.

## Data Availability

The data analyzed in this study is subject to the following licenses/restrictions: The raw sleep EEG data supporting the conclusions of this article will be made available by the authors upon reasonable request, without undue reservation, to qualified researchers. The public MEG datasets analyzed during this study are available through the Human Connectome Project (HCP) repository. Requests to access these datasets should be directed to Maria Corsi-Cabrera, corsi@unam.mx.
